# Progress of Veterinary Science

**Published:** 1883-01

**Authors:** 


### Progress of Veterinary Science.

*Erratum*—On page 54, 11th line, after 3 insert ½.

Heredity.—Brown Sequard, at a meeting of the French Academy of Sciences, March 13, 1882. presented a communication on experiments made on Guinea pigs, which prove that accidental affections of the parent are sometimes transmitted to the offspring.

During his researches Mr. Brown-Sequard has proved the possibility of introducing a tube into the larynx of the higher animals without causing any pain or any subsequent bad result, although the experiment was performed repeatedly, in at least one case, on a single subject. The local insensibility to pain was caused by directing a rapid current of carbonic acid upon the upper part of the larynx through an incision, for from fifteen seconds to two or three minutes. After the operation was completed the anæsthetic effect lasted from two to eight minutes.

Diseased Fowls.—According to a yearly report of *post mortem* examinations sent to the (English) *Live Stock Journal*, more deaths are due to diseased liver than to any other cause. Out of a total of 343 cases in 1881 only 9 died from roup, while 85 succumbed to a diseased liver and 52 to inflammation of the bowels.

Bronchitis in Birds.—Mix a piece of stale bread with an equal quantity of the coarsest brown sugar, moistened with water, and give it to the bird.—*Land and Water.*

A Good Medicine for Poultry.—One gill of good whiskey, one-third of a gill of water, one teaspoonful of Harvell's condition powders, one-half teaspoonful cayenne pepper, and mix well together. Give one teaspoonful of the mixture every three hours. If the fowl is very sick physic lightly with castor oil. The above is a sure cure forany disease arising from cold if taken in time.—*Poultry Bulletin.*

A Bacillus or a Fat-Crystal.—At a meeting of the Pathological Society at the Charity Hospital, New Orleans, November 21st, an important microscopic demonstration was made by Dr. H. D. Schmidt, President of the Society. Dr. Schmidt stated that beyond doubt the bacilli were fat-crystals. Dr. Schmidt succeeded in finding crystals, which were similar in appearance to bacilli discovered by Koch, and apparently the same. To determine their nature, Dr. Schmidt subjected the crystals to the action of boiling ether, when they disappeared, proving, as he claims, that they were not germs or organisms.

The Pepsin of Sheep.—M. Gautier has shown that sheep pepsin, well prepared, contains about two per cent. of its weight of insoluble granules, which can be collected and washed on filters of porous porcelain, and appear then to consist of refracting corpuscles rounded or ovid in shape. In spite of their insolubility these corpuscles seem to possess considerable digestive power, since they can peptonise albuminous bodies, and if left for a long time in pure water, or in water which is slightly acidulated, the substance of which they consist becomes slowly transformed into a soluble pepsin.

BIRDS AND TRICHINÆ.—Birds are not susceptible to infection by trichinæ spiralis. They may be fed upon trichinous pork with impunity.

HYDROBROMATE OF HOMATROPINE has been found very useful for rapidly dilating the horse's pupil. *Archir f. Threrheilkunde.* It acts more quickly than atropia and its effects disappear more rapidly.

A STRIKING CASE OF ELEPHANTIASIS IN A HORSE is related by Drs. Lustig and Rabe in the jahresberecht der Kong Thierarz, zu Hanover. The disease affected chiefly the lower jaw and right fore leg. The condition after death was, varicose elephantiasis of lower jaw and each carpal joint.

SOFTENING OF BONES IN THF COW.—Mollities orsium in the cow is almost always cured, says Dr. Harris by the use of hydrochloric acid. He gives about ℥ ii. of concentrated acid in a pint of water, from three to six times a day.

THE HORSE FAMILY.—The horse family (Equidæ), the horse genus (Equus), and the horse species (*E. Caballus*) are often confounded in the popular mind. It may not be amiss, therefore, to sketch briefly the geological history of the family. Introduced first in Early Tertiary, the family was represented in Eocene, Miocene, and Early Pliocene only by polydactyle forms, in which, however, a gradual increase in the middle hoof and diminution of the side hoofs may be traced. In the Upper Pliocene was first introduced a single-hoofed form (Pilohippus,) which, however, became extinct at the end of the Pliocene and did not enter the Quaternary. The horse (genus Equus) was apparently introduced first in the Upper Pliocene and ranged through the Quatenary in successive species: *E. occidentalis, Pacificus, major*, and finally, *Caballus*. These all became extinct at the end of the Quatenary, and the last (viz., *Caballus*) was reintroduced on this continent in historic times.

ASTRAGALUS MOLLISSIMUS.—Among the plants destructive to cattle in the West (Weis Remedies, 1882), is the Astragallus Mollissimus, whose physiological action has recently been studied by Dr. Isaac Ott, of Easton, Pa. He summarizes it as follows: It decreases the irritability of the motor nerve; greatly affects the sensory ganglia of the central nervous system, preventing them from readily receiving impressions. Has a spinal tetanic action. It kills mainly by arrest of the heart. Increases the calloary secretion. Has a stupefying action on the brain. Reduces the candiac force and frequency. Temporarily increases arterial tension, but finally decreases it. Greatly dilates the pupil.—*Chicago Medical Review.*

PARASITIC DISEASE IN A DOLPHIN.—M. Meguin presented to the Societie de Biologie recently a very much afflicted dolphin. In its stomach were ascarides (*ascaris simplex*); in the biliary ducts were a number of filliform nematoids strongly embedded in the mucous membrane. Finally in the muscular tissues were numerous cystic worms resembling echinococci.

DISEASES AND ACCIDENTS AMONG OSTRICHES.—Few birds, when properly cared for, die from any sickness; more are killed by accident, the most common of which is the breaking of a leg. Even this, however, is rare, and with ordinary attention the risk of mortality with this hardy bird is very small. When frightened during the night they may run at full speed over broken country, yet almost invariably escape uninjured. The reason appears to be that their wings bear them up, and they touch the ground quite lightly. If one breaks a leg, it happens in the day-time, when carefully stepping over a stony place, with wings at rest; the foot slips, or a sudden fright causes the fall, and hence the accident. But as we have said, such rarely occur.

THE NERVOUS DISEASES OF PACHYDERMS.—This class of animals are very rarely subject to any form of nervous disease. The elephant has cerebral congestions and occasionally meningitis, but has few other nervous disturbances. A case of posterior paralysis in the tapir and another in the hippopotamus have been observed. Occasionally the cysticercus cellulosæ gets into the brain of *sus scrofa* and causes trouble. Dr. Max Schmidt in a review of the diseases of pachyderms was able to collect but very few instances of nervous disease.

CRANIAL CAPACITY OF THE HORSE.—Dr. F. Eichbaum of Giessen (*Archiv fur W. und P. Thierheilkunde*) has made some elaborate investigations regarding the size of the horse's skull.

The following cranial capacities are given :

| | Cranial capacity. |
|---|---|
| Arabian horse | 712 Cubic c. m. |
| Russian horse | 720 " " |
| Belgian horse | 768 " " |

Eichbaum finds :

1. That the cranial part of the skull is more developed in proportion to the anterior or facial part of the skull in eastern than in western (European) horses.

2. The breadth of the skull of eastern horses is greater in proportion to length than that of western horses.

INCUBATION OF RABIES IN THE DOG—The most valuable evidence on this point was published some years ago by the Medical Press Commission, and from its reports we gather that the period of incubation in animals is very uncertain. In many cases it cannot be ascertained with any degree of probability, as animals are frequently wounded by others without any particular notice being taken of the circumstance; and also as frequently without anyone observing the injuries at the time of or after their infliction. Indeed, it is common to hear owners of rabid dogs assert that their dogs have never been from home, or, if abroad. have never left them for a second; that they could not therefore, be bitten without their knowledge, and they were not cognisant of such an occurance. The evidence of statistics and facts may be useful in fixing an approximate time. La Fosse states that the shortest authenticated period in his experience was seven days, and the longest one hundred and fifty-five days. Roll gives from three to six, and rarely from seven to ten weeks; whilst Blain asserts that the majority of cases occur between the third and seventh week, though some are protracted to three, four, or even a greater number of months. A week was the shortest period he had noticed. Youatt has known instances in which the first symptoms have only become manifest after from five to seven months, and he never knew of a case occuring before seventeen days intervening. Of nine cases which Bench could rely upon, the symptoms appeared after the bite in each, at an interval of ninety-five, eighty-eight, thirty-five, twenty-six, twenty.four, twenty-two, eighteen, fifteen, and ten days respectively. In 1867 Renault reported that of sixty-eight dogs inoculated experimentally or bitten the malady was developed in :—

| | | | | | |
|---|---|---|---|---|---|
| 1 | from 5th to 10th day | 2 | from 35th to 40th day | 4 | from 60th to 65th day |
| 4 | " 10th " 15th " | 3 | " 40th " 50th " | 1 | " 65th " 70th " |
| 6 | " 15th " 20th " | 7 | " 45th " 50th " | 4 | " 70th " 75th " |
| 5 | " 25th " 30th " | 2 | " 50th " 55th " | 2 | " 80th " 90th " |
| 10 | " 30th " 35th " | 2 | " 55th " 60th " | 1 | " 100th " 118th " |

In Saint Cyr's cases of confirmed rabies, 1865, there were only twenty-six the date of whose inoculation could be positively ascertained. In these the latent period was :—

| Cases. | Days. | Cases. | Days. | Cases. | Days. |
|---|---|---|---|---|---|
| 1 | 16 | 2 | 32 | 2 | 50 |
| 1 | 18 | 1 | 33 | 2 | 60 |
| 3 | 21 | 1 | 35 | 1 | 62 |
| 2 | 24 | 1 | 36 | 1 | 86 |
| 1 | 30 | 1 | 38 | 2 | 90 |
| 1 | 31 | 1 | 41 | 2 | 105-115 |

Bouley has known instances in which the latent period was twelve days to seven months, though they were rare. It was uasually from six to twelve weeks. According to Haubner, in two hundred cases the appearance of the disease within two months was eighty-seven per cent.; three months, sixteen per cent.; four months, one per cent. He mentions an instance in which the incubation period was from seven to eight months, and another in which it was fourteen months. From these statistics and facts it may be seen that nothing can be said with certainty or in a positive manner as to the length of the interval which elapses between the receipt of the injury and the appearance of rabies, and that it is therefore hazardous to say when a dog has been wounded by one that is rabid at what period it may be considered safe from au attack of the disease, as there are no reliable limits to this latent period. —*Land and Water.*

Recovery from Rabies.—On more than one ground the possibility of the recovery of dogs from attacks of rabies is of great importance. The demonstration that this terrible disease is not invariably fatal, even in the animals most prone to it, may at least be welcomed as affording a ray of hope for therapeutics, while the fact of the recovery of the affected animals may afford an explanation of many mysterious outbreaks of the disease. M. Decroix latelycommunicated to the Academie de Medecine nine cases which he had collected of well-authenticated recovery from rabies. Decroix points out that in furious rabies the attacks increase in frequency and intensity during two or three days, then attain their maximum, and disappear in two or three days more whereas death does not occur until the fifth or sixth day. The rabies commission in France has made a host of experiments with various substances of reputed value in rabies, three of them with pilocarpine, and every supposed remedy which they employed appeared actually to hasten death by the violent paroxysms which it caused. The conclusions of M. Decroix are that it is experimentally demonstrated that rabies may terminate in spontaneous recovery. Up to to the present day no agent has made good its claim as a remedy for rabies.

The cases of recovery attributed to this or that agent may be, with equal justice, ascribed to the spontaneous termination of the disease. In absolute quietude and obscurity the paroxysms are far less terrible than when medicines are administered, and M. Decroix asserts that if these conditions could be secured, he would far rather suffer from hydrophobia than from many other diseases. It may however be observed that we are scarcely justified in drawing, from the superior results of therapeutic inactivity in dogs, the same lesson in the case of the disease in man. It is very desirable, in the case of any recovery in rabies, that it should be ascertained at what rate the saliva ceases to be infectious, and whether the disease can be communicated after the animal has to all appearance recovered. This is not an improbable explanation of the occasional alleged occurence of the disease from the bite of healthy animals.—*London Lancet.*

A Case of Hydrophobia treated successfully with Aconite.—Dr. Cullimore, physician to the Northwest London Hospital reports, a case successfully eured with aconite. The treatment, after placing the patient in a quiet and secluded corner, consisted of a dietary of milk thickened with arrowroot and beef, with the following mixture: one minim of the tincture of aconite, six grains of bromide of potassium, six minims of the tincture of cinchona, to half an ounce of water, to be taken every half hour for twelve doses, and then three times a day.

Interesting to Veterinary Dissectors.—*Semper's Method of Making Anatomical Preparations.*—Dr. Semper has discovered a method of making anatomical preparations which assures the preservation of the several organs. He reports the result of his work in the British Medical Journal. He has used the method constantly for several years, and the results are said to be excellent. After the dissection is made, it is macerated from one to five days, according to its proportions, in weak chromic acid (one to two per hundred), it is then washed, and treated with weak alcohol (thirty per hundred), adding alcohol of gradually increased strength. The preparation is then placed in strong alcohol from one to seven or eight days; afterwards in absolute alcohol, and finally is left to macerate in terpentine until it is thoroughly soaked. The preparation is then spread out and allowed to dry by contact with the air, it thus becomes perfectly white. The different organs can be colored by using the best oil colors. When the preparations have been thoroughly dried after their maceration in turpentine, they resume to a great extent their natural coloring if they be immersed in a mixture composed of equal parts of a saturated solution of white sugar and glycerine.

In the same connection we would refer to Dr. Roswell Parks' method of making

Flexible Anatomical Preparation of Joints.—The joint to be prepared should be carefully dissected by aid of maceration so as to remove thoroughly all the soft parts except the ligaments. It may then be bleached by hydrochlorous acid in the following way: a small quantity, four to five grammes, of powdered potassium chlorate is put in a stone jar, and 20 cc. of strong hydrochloric acid poured over it. The jar is then filled with water and the specimens dropped into it. From six to thirty hours in this solution suffice. After still further scraping and cleaning they are finally placed in the following mixture:

| | | |
|---|---|---|
| Coffee sugar | 2 | parts. |
| Saltpetre | 1 | " |
| Methylic alcohol | 1 | " |
| Glycerine | 16 | " |

A little water may be used to assist in the solution of the solids, or a good article of syrup may be substituted for the sugar. A little thymol may also be added with advantage, though it is not necessary it should be dissolved in the alchol.

In this mixture the specimens are allowed to remain from one to two or even three weeks, according to their size. After removal from it they are allowed to drain for a few days, and then need only a little trimming and scraping before being placed in the cabinet.

Intestinal Disorder in Horses.—Most Horseman have, at some time or other, experienced the annoyance incidental to the possession of a horse which was afflicted with what is commonly termed looseness of the bowels. The derangement does not amount to an attack of diarrhœa, which might be met by appropriate remedies and cured; but, at all sorts of irregular and particularly inconvenient periods, the animal voids a large quantity of dung which is mixed with a good deal of fluid, and generally has an extremely fœtid odour. It is commonly remarked of horses which are subject to attacks of looseness of the bowels without any tangible cause, that what they eat seems to go through them without doing them any good; and such animals are seldom in very good condition, but are tucked up and miserable in appearance, and after any active exertion assume a forlorn aspect, which disgusts the owner, and perhaps causes him to use strong language to his groom.

There can be no doubt that in all cases, whatever may be the kind of diet on which the animal is kept, there is an excessive irritability of the mucous membrane of the digestive system.

Occasional attacks of looseness of the bowels under special circumstances, as, for example, the excitement of the chase, indicate a high degree of nervous irritability: but, although rather annoying to the rider, they do not injure the animal, and there is some satisfaction in knowing that nothing can be done in the way of medical or dietetic treatment to mitigate the disorder, so long as the animal is exposed to the excitement on which it depends. It is best, therefore, in such cases, to put up with the inconvenience and let the animal take its chance.

"Looseness" arising from chronic irritability of the lining membrane of the digestive canal is in fact a form of indigestion; and the great portion of the food consumed is, in the peculiar state of the animal, so much indigestible substance, which the stomach and intestines reject as quickly as possible.

By the term "indigestible matter" it is not intended to suggest that the food is necessarily of bad quality, as the oats, hay, bran, and beans may be quite up to the average, and constitute digestible food for horses whose digestive organs are in good order; but it is clear, from the results of a meal, that such diet in the case of the "washy" horse acts as an irritant, and is not retained long enough for the digestive process to be completed, or even carried to an advanced stage.

Medicine cannot be expected to effect much good in the form of indigestion to which we have referred. All that can be done in the way of cure must be effected by strict attention to diet.

At the commencement of the treatment, a dose of linseed oil will clear out the intestinal canal and get rid of any irritating matters which keep the membrane in an excitable state; and, after the laxative has produced its effect, a perfectly bland, non-irritating diet may be tried and continued for some time, in the hope that the mucous membrane may recover its normal tone. Milk and eggs are most easily obtained, and form, together or separately, a perfectly digestible and highly nutritious kind of food, of which a horse will soon become fond, and which he will take without hesitation. The quantity of the food to be given daily will depend on the horse's condition and appetite. Two quarts of milk, with three or four eggs, three times in the day, will be ample for the support of the system; and if it be intended to attempt a radical cure of the disease, it will be best to keep away all solid food for a time.

Instead of milk and eggs, soup made of coarse pieces of meat may be used, and experience has shown that emaciated animals have improved in condition rapidly on such fare. A total change in the system of dieting will generally be too serious an innovation to be attempted in the owner's stable; and, unless he is an enthusiast on the subject, and determined to carry out the experiment at all hazards, it is not likely to be put in practice at all; but there is no question of the curative value of a dietetic system which consists in the substitution of a non-irritating diet for the food which is in ordinary use.

When the expedient of a total change of diet for the cure of looseness of bowels cannot be tried, there is little to be done beyond regulating the ordinary diet, avoiding beans and green food, and trusting to good old hay, with occasionally linseed mashes, and take special care not to allow the horse to take a large draught of water immediately before eating. To prevent this, it is better to keep water always before the animal. Medicine, we have said, cannot be expected to do much: but, when the ordinary kind of diet is continued, it may be worth while to try the effect of small doses of aloes with gentian and salts of iron. A dose of half a drachm of Barbadoes aloes, with a drachm of powdered gentian and twenty grains of sulphate of iron, may be made into a ball and given once a day for some time, unless a too energetic action on the bowels results, in which case the medicine would be discontinued for a few days. At the best, it must be allowed that horses which are liable to the disease in question are objectionable animals to deal with, and the experience of the most carefully conducted treatment is not encouraging.—*Field.*

Comparative Dietetics.—Under this heading, perhaps not happily chosen, I beg to offer you an account of a peculiar case in a dog treated by me. It was a small black-and-tan terrier, and the symptoms were as follows: Great weakness and depression, vomiting and convulsions. The dog had shown all these symptoms except the convulsions for two days when I first saw it. There was a total loss of appetite, and convulsions followed immediately upon any food being introduced into the stomach. There was no diarrhœa, and my diagnosis was that some mechanical irritant had been swallowed. To allay the gastric irritation small doses of hydrocyanic acid were given. To combat the depression a little brandy was mixed with a solution of Brand's essence of beef, and one or two teaspoonfuls given occasionally. Marked improvement followed. The brandy was discontinued and the beef essence used as food. In two days such was the improvement—no return of either convulsions or vomiting—that a little milk was tried, but vomiting was speedily followed, and the essence was resumed. Again the improvement was noticeable, and as the stomach seemed calm a little pounded raw steak was given. Vomiting returned and before it was ejected a slight convulsion occurred. Being convinced that some irritant remained in the stomach, aperients were resorted to with a hope that it might pass on into the intestines. Emetics were not used, as it was thought that the vomiting already induced would have accomplished any removal which could have been affected by them. The dog died on the eighth day, having been kept alive entirely on the essence of beef, of which it consumed four small tins. A post-mortem examination showed a small phial cork firmly wedged into the pyloric opening of the stomach, effectually preventing anything passing into the intestines. There was nothing else in the organ and no disease of any other part. The interest to me is the fact that not only solid food, but soup, milk and ordinary beef tea caused vomiting and frequently vomiting and convulsions, whilst the essence of beef was followed by neither, but was absorbed directly through the stomach. Since this case I have often used the same article in irritable conditions of the stomach and with the best results. The case, I think, also demonstrates what my practice bears out—that milk, supposed to be a bland and inoffensive article of diet, is very injurious in cases where the mucous membrane of the stomach and intestines is excitable or weak.—*London Lancet.*

THE INFLUENCE OF ANIMAL FOOD ON THE PRODUCTION OF WOOL.—The *Journal de Medecine Veterinaire* for September contains an article by M. P. Regnard on the effects of an animal diet on herbivora, in substitution for the ordinary vegetable provender. The writer remarks at the beginning that numerous attemps have been made to nourish herbivora with animal food, with doubtful success in most cases; but he suggests that the mode of procedure was far from economical, and was, therefore, not likely to be popular.

In spite of the bad results which had attended experiments in alimentation, M. Regnard was led to believe that, if animals were from the time of birth kept on animal diet of a highly azotised kind, the effects on the growth of the tissues must be advantageous; and, in support of the idea, he refers to the fact that in the early periods of their existence all herbivora are purely carnivorous in their feeding, inasmuch as they subsist entirely on the milk of the dam.

Reasoning from the above facts, it was natual to inquire whether there was a possibility of replacing the natural food, the milk of the mother, by some other highly nutritive animal substance; and the investigator at once selected the blood as a substance which could be used economically for the purpose, there being no lack of the material in the abattoirs. Blood in its crude state cannot be used as food for herbivora—in fact, the animals refuse to touch food in which the fluid is mixed; but, after being heated and the clot subjected to pressure, it can be rapidly dried, and then ground to a fine powder, which is free from odor or taste, not prone to decomposition and unaffected by being mixed with any kind of provender.

Experiments with the prepared blood were tried on some lambs in an emaciated state; while, in order to compare the system with the ordinary plan of feeding, other lambs were fed in the usual way. It was found that the animals which were fed on the cooked and dried blood improved much more in condition than did those which took the ordinary diet, and the verdict of the farmers of the district was that such fine lambs of the age had never before been seen. Experiments in the above direction are being tried on calves; and the author suggests that, if they succeed, it is impossible to overrate their economic value, as the feeding of calves for the butcher or for other purposes will no longer necessitate the sacrifice of the milk of the cow, which may be employed in the dairy without detriment to the young animals.

In reference to the influence of cooked and dried blood in the production of wool, M. Regnard gives the results of the feeding of lambs of different breeds, and it would appear that those which were nourished on ordinary food fell far below the others in regard to the weight of their fleeces. Three lambs which were fed in the usual way gave together 555 grammes of wool when shorn, and three of the same age fed on dried blood gave 1060 grammes, or nearly double the quantity.

Whether or not any practical consequences will result from M. Regnard's experiments, there is no doubt of their scientific value; and, as he remarks, if the system of feeding on dried blood were generally adopted, there would at once be established a remunerative use for the vast quantities of blood which are wasted in the abattoirs. We may add also that, as soon as its value was recognized, blood would cease to be a waste material, and would command its full value in the market.

INDUCED PREDISPOSITION TO ANTHRAX.—The temperature most favorable to the development of the bacteria of anthrax is that of the mammalia—*i. e.*, a temperature of 37° or 38° C. Birds and especially fowls, which have a higher temperature (42°) do not, under ordinary conditions, contract the disease. M. Pasteur, however, as is known, succeeded in communicating the disease to fowls, and in causing the bacteria to develop in their blood by lowering the body heat by means of the immersion of one foot for a considerable time in cold water. In the case of frogs, it was found by M. Gibier that at the ordinary temperature of water they are unaffected by a subcutaneous or intra-peritoneal injection of the liquids of anthrax, and it became important to ascertain whether, by raising the temperature to 37°, and thus render them for a time warm-blooded animals, the bacteria of anthrax would develop in their blood. Experiments, directed to this end, afford at least a partially affirmative answer. By compelling frogs to live in water at a temperature of from 35° to 37° C., M. Gibier succeeded in communicating the disease to them. The experiment does not always succeed, even when the conditions are the same. Of twenty animals the disease was communicated to five only. The others died as soon as they were immersed in warm water, or in the course of three or four days without presenting evidence of injection. Two, moreover, resisted entirely. The blood of the other five, however, presented abundant bacteria, and there were also found in great numbers in the liver. A drop of blood taken from the heart of one and inoculated under the skin of a guinea-pig killed it in forty-eight hours. The bacteria in the frog were remarkable for their length, which was far greater than that of the bacteria of the guinea-pig. This, M. Gibier attributes to the slowness of the bacterian circulation, conjecturing that in the rapidly moving blood ot the mammalia the rods may be broken. Some other points were also noticed in the course of these experiments. It was observed that the frogs which proved susceptible had fasted for a longer or shorter time. Young, vigorous, newly-captured frogs either resisted altogether or died at the end of two or three days without any development of bacteria or increase in the size of the liver. Frogs which were suddenly removed from cold to hot water, without any gradual transition, as soon as possible after the inoculation, died more quickly from the disease than did those which had been acclimatized by degrees. Lastly, a curious fact was noted, that none of the frogs which had been previously inocculated in the cold water were affected by a previous inocculation in warm water. This may have been only an accidental coincidence, but it may possibly have a vaccination phenomenon." —*London Lancet.*

ENGLISH VETERINARY NEWS.—The *Gazette*, of November 17, publishes the names of a crowd of officers who are decorated and promoted for service during the late campaign in Egypt, and among them appears the name, for the first time, of an officer of the veterinary department—J. J. Meyrick, Inspecting Veterinary Surgeon. He received the companionship of the Bath (C. B.) and the third class of the Osmanieh—an Egyptian or Turkish distinction.

Veterinary Surgeon F. F. Walker, First Life Guards, with other selected officers and men, attended at Windsor Castle, to receive the Egyptian War Medal from the Queen in person.

On the recommendation of the Director-General of Military Education, a new appointment—that of Veterinary Lecturer—has been added to the staff of the Department of Artillery Studies at Woolwich. First class Veterinary Surgeon J. C. Dwyer, has been selected for the part, the creation of which has been long thought desirable.—*Veterinary Journal.*

